# Validation of a Commercial Insulated Isothermal PCR-based POCKIT Test for Rapid and Easy Detection of White Spot Syndrome Virus Infection in *Litopenaeus vannamei*

**DOI:** 10.1371/journal.pone.0090545

**Published:** 2014-03-13

**Authors:** Yun-Long Tsai, Han-Ching Wang, Chu-Fang Lo, Kathy Tang-Nelson, Donald Lightner, Bor-Rung Ou, Ai-Ling Hour, Chuan-Fu Tsai, Cheng-Chi Yen, Hsiao-Fen Grace Chang, Ping-Hua Teng, Pei-Yu Lee

**Affiliations:** 1 Department of Research and Development, GeneReach Biotechnology Corporation, Taichung, Taiwan; 2 Institute of Biotechnology, National Cheng Kung University, Tainan, Taiwan; 3 Institute of Bioinformatics and Biosignal Transduction, National Cheng Kung University, Tainan, Taiwan; 4 Department of Veterinary Science and Microbiology, University of Arizona, Tucson, Arizona, United States of America; 5 Department of Animal Science and Biotechnology, Tunghai University, Taichung, Taiwan; 6 Department of Life Science, Fu-Jen Catholic University, Taipei, Taiwan; Naval Research Laboratory, United States of America

## Abstract

Timely pond-side detection of white spot syndrome virus (WSSV) plays a critical role in the implementation of bio-security measures to help minimize economic losses caused by white spot syndrome disease, an important threat to shrimp aquaculture industry worldwide. A portable device, namely POCKIT™, became available recently to complete fluorescent probe-based insulated isothermal PCR (iiPCR), and automatic data detection and interpretation within one hour. Taking advantage of this platform, the IQ Plus™ WSSV Kit with POCKIT system was established to allow simple and easy WSSV detection for on-site users. The assay was first evaluated for its analytical sensitivity and specificity performance. The 95% limit of detection (LOD) of the assay was 17 copies of WSSV genomic DNA per reaction (95% confidence interval [CI], 13 to 24 copies per reaction). The established assay has detection sensitivity similar to that of OIE-registered IQ2000™ WSSV Detection and Protection System with serial dilutions of WSSV-positive *Litopenaeus vannamei* DNA. No cross-reaction signals were generated from infectious hypodermal and haematopoietic necrosis virus (IHHNV), *monodon* baculovirus (MBV), and hepatopancreatic parvovirus (HPV) positive samples. Accuracy analysis using700 *L. vannamei* of known WSSV infection status shows that the established assayhassensitivity93.5% (95% CI: 90.61–95.56%) and specificity 97% (95% CI: 94.31–98.50%). Furthermore, no discrepancy was found between the two assays when 100 random *L. vannamei* samples were tested in parallel. Finally, excellent correlation was observed among test results of three batches of reagents with 64 samples analyzed in three different laboratories. Working in a portable device, IQ Plus™ WSSV Kit with POCKIT system allows reliable, sensitive and specific on-site detection of WSSV in *L. vannamei.*

## Introduction

White spot syndrome, an OIE-listed disease, has resulted in severe economic losses in the shrimp aquaculture industry worldwide [1]. The disease is caused by white spot syndrome virus (WSSV), an enveloped large double-stranded DNA virus belonging to genus *Whispovirus* in the family *Nimaviridae*. Late post-larvae, juvenile and adult penaeid shrimp could all be infected by WSSV both vertically and horizontally. WSSV replicates preferentially in the cuticular epithelium and subcuticular connective tissues, as well as in the antennal gland, and haematopoietic tissues in shrimp. Factors such as rapid salinity and temperature changes can help activate WSSV in apparently healthy carriers and trigger the onset of the disease. Once activated, the disease could progress quickly and causes 100% mortality within 5 days [2], [3]. In addition, WSSV could be found in a broad range of freshwater and marine crustaceans, including shrimp, crabs, crayfish and lobsters [4], [5], [6]. Pre-screening of WSSV-free broodstock or larvae and regular surveillance of WSSV infection are important strategies to prevent WSSV infection outbreaks. Samples from pleopods, gills, haemolymph, stomach or abdominal muscle are recommended for diagnostic testing in shrimp [2].

Critical biosafety measurements could be implemented effectively when pond-side WSSV detection could be carried out to provide rapid, sensitive and specific test results. Polymerase chain reaction (PCR)-based assays, including basic PCR, nested PCR, and real-time PCR [7] assays, have been developed and demonstrated to be highly sensitive and specific for WSSV detection. However, these assays generally are not suitable for on-site applications because they require a trained technician and a relatively complicated thermocycler to operate. Furthermore, risks of cross contamination are relatively high due to the requirement of post-amplification processing of the reaction to detect PCR products for all PCR assays except for real-time PCR, which nevertheless requires a relatively expensive device for signal detection and processing.

Recently, insulated isothermal PCR (iiPCR) was developed successfully to work in a simple thermally baffled device [8]. Within a specially designed cylindrical vessel (R-tube™; GeneReach Biotech, Taichung, Taiwan), iiPCR works by cycling reactions through temperature gradients established by the Rayleigh-Bénard convection when the tube is heated at the bottom with a single heating source. The three PCR steps, namely denaturation, annealing and extension, can be completed at different zones within the vessel. A WSSV iiPCR assay was reported to successfully produce detectable amplicons in 30 minutes with notable sensitivity and specificity [9]. However, the requirement of gel analysis to detect amplicons led to high risks of the assay amplicon cross-contamination, which was eliminated subsequently by incorporating hydrolysis probe technology into iiPCR and integration of an optical detection module into the iiPCR device [10]. An iiPCR device, namely POCKIT™, is now commercially available to perform the fluorescent probe-based iiPCR and provide test results automatically.

Taking advantage of the POCKIT system, IQ Plus™ WSSV Kit with POCKIT system was developed for on-site detection of WSSV in shrimp samples. Following procedures described in OIE Validation and Certification of Diagnosis Assays [11], the assay was evaluated and validated for WSSV detection in *Litopenaeus vannamei*. We report results for the assessment of analytical sensitivity, analytical specificity, repeatability, diagnostic accuracy, and proficiency of the established assay.

## Materials and Methods

### Sample Collection

*L. vannamei* samples were purchased from shrimp farms in Taiwan. All shrimp were shipped in oxygen-filled bags to GeneReach’s lab and maintained for at least one day before screening. For experimental infection, a WSSV isolate obtained from naturally infected *Penaeus monodon* in Taiwan in 1994 was used. The status of WSSV infection in *L. vannamei* was defined by both an OIE reference method [5] and OIE-registered WSSV diagnostic kit, IQ2000™ WSSV Detection and Protection System (DPS). Infected shrimp were stored at −20°C before further analysis.

For proficiency evaluation of IQ Plus™ WSSV Kit with POCKIT system, abdominal muscles of *L. vannamei* experimentally infected with WSSV were aliquoted and preserved in 95% ethanol. A total of 31WSSV-negative and 34 WSSV-positive specimens were selected by IQ2000™ WSSV DPS. Aliquots of these samples were mailed to the laboratories of Dr. Chu-Fang Lo (National Taiwan University, Taiwan), Dr. Bor-Rung Ou (Tung-Hai University, Taiwan), and Dr. Donald V. Lightner (University of Arizona, Tucson, USA) and tested by two operators at each site.

### Nucleic Acid Extraction

DNA was extracted by using IQ Plus™ Extraction Kit (GeneReach Biotech) as described in the user manual. Briefly, tissues (50 mg) of ectodermal and mesodermal origin (e.g. abdominal muscle, pleopods, or periopods) were ground thoroughly in 500 µl of Solution 1. After the addition of Solution 2 (500 µl), the mixture was centrifuged at maximum speed using a cubee™ (GeneReach Biotech) at room temperature for 1 min. Supernatant (500 µl) was added to the spin column and centrifuged at maximum speed at room temperature for 1 min. The spin column was washed once with 500 µl of Solution 2 before DNA was eluted with 200 µl of Solution 3.

### Preparation of WSSV Genomic DNA

Purified WSSV virions were prepared as described [12]. The concentration of purified DNA was determined by UV spectrophotometry and calculated on the basis of published genome size of 307,287 bp (GenBank accession no. AF440570).

### IQ Plus™ WSSV Kit with POCKITsystem

The reaction in POCKIT™, an iiPCR-compatible instrument, completed a run in about one hour with the test readouts displayed on the monitor. Probe signals were collected during the reactions without the need to open the reaction vessels. POCKIT™ was developed to include two separate channels, *i.e.* wavelengths 520 nm and 550 nm, on the basis of the optical detection module described previously for the original iiPCR device [8], [10]. Signals passing through the 520- and 550-nm channels were collected by an integrated circuits controller-regulated CMOS sensor. Signal-to-noise (S/N) ratios were calculated by dividing light signals collected after iiPCR by those from before iiPCR [10]. Limit of blank and positive cut-off for POCKIT™ were assigned on the basis of S/N ratios of numerous NTC (non-template control) and positive iiPCR reactions for various targets according to the reference method [13] and validated by analytical specificity and sensitivity tests (data now shown). Based on default thresholds of S/N ratios, results were converted automatically to “+”, “−“, or “?” and shown on the display screen. A “?” result indicated that the signals were ambiguous and the sample should be tested again.

Based on iiPCR and fluorescent probe hydrolysis for signal detection [10], IQ Plus™ WSSV Kit with POCKIT system (GeneReach Biotech) was designed to work in POCKIT™. Target-specific PCR primers and probe for WSSV were designed to interact with a segment of the *major capsid protein* gene of WSSV. To monitor DNA extraction and iiPCR reactions, primer pair and probe targeting shrimp nuclear 18S rRNA gene were included to serve as the internal control (IC). All primers, probes, and dNTPs were provided in a lyophilized Premix pellet. To assemble the reaction, the Premix pellet was first rehydrated in 50 µl of Premix Buffer B. After 5 µl of extracted DNA were added to the Premix mixture, 50 µl of the final reaction mixture were transferred to an R-tube™ (GeneReach Biotech). The R-tube™ was sealed with a cap, spun for 10 sec in a cubee™ (GeneReach Biotech), and placed into a POCKIT™. The reaction was completed in one hour. Target and IC signals were collected through the 520- and 550-nm channels, respectively.

### Nested PCR

IQ2000™ WSSV, IHHNV, MBV and HPV DPSs (GeneReach Biotech) were used to screen shrimp samples for the presence of target pathogens. Reactions were assembled and carried out as described in the manual. Briefly, the program for the first WSSV PCR included denaturation at 94°C for 2 min, 15 cycles of 94°C for 20 sec, 62°C for 20 sec and 72°C for 30 sec, and a cycle of 72°C for 30 sec and 20°C for 20 sec. The second PCR included 30 cycles of 94°C for 20 sec, 62°C for 20 sec and 72°C for 30 sec and one cycle of 72°C for 30 sec and 20°C for 20 sec. Nested PCR products were analyzed by electrophoresis on 2% agarose gels and stained with ethidium bromide.

### Statistics

Statistical probit analysis, a non-linear regression model, was performed using commercial software SPSS 14.0 (SPSS Inc., Chicago, Illinois, USA) to determine limit of detection (LOD) with 95% confidence. Kappa statistic test (κ) was used to test the level of agreement between two different assays. Test results from different laboratories and operators in proficiency studies were analyzed by Chi-squire test and Fisher’s exact test (for numbers less than 5) for homogeneity.

## Results

### IQ Plus™ WSSV Kit with POCKIT System has Sensitivity Similar to Nested PCR

The recently developed POCKIT™ device is designed specifically to provide optimal conditions for fluorescent probe-based iiPCR. The device has the capacity to detect 520-nm and 550-nm signals simultaneously to detect multiplex signals. Taking advantage of the POCKIT system, a multiplex WSSV assay (IQ Plus™ WSSV Kit with POCKIT system) that could detect WSSV DNA and a shrimp IC target simultaneously was developed. Inclusion of an IC in the assay helps control for DNA extraction and subsequent enzymatic reactions.

First, to evaluate analytical sensitivity of IQ Plus™ WSSV Kit with POCKIT system, 10-fold serial dilutions of either linearized WSSV standard plasmid (pWSSV1) or WSSV genomic DNA of known copy numbers were analyzed. Analysis of 16 replicates of each dilution of standard plasmid shows that 100%, 43.75% and 18.75% of the 10^2^-, 10^1^-, and 10^0^-copyreactions, respectively, produced positive WSSV (520 nm) signals (Table 1). The LOD [95% hit rate] determined by probit regression analysis for standard plasmid was 24 copies per reaction (95% confidence interval [CI], 16 to 70 copies per reaction). Similarly, positive signals were generated from 100%, 62.5% and 25% of the reaction containing 10^2^, 10^1^ and 10^0^ copies of WSSV genomic DNA, respectively. The LOD determined by probit analysis with WSSV genomic DNA was17 copies per reaction (95% confidence interval [CI], 13 to 24 copies per reaction).

**Table 1:** Assessment of analytical sensitivity of IQ Plus™ WSSV Kit with POCKIT system.

Copies/reaction	Template	
	pWSSV1*	WSSV genomic DNA
10^3^	100.00% (16/16)	100% (16/16)
10^2^	100.00% (16/16)	100% (16/16)
10^1^	43.75% (7/16)	62.50% (10/16)
10^0^	18.75% (3/16)	25.00% (4/16)
10^−1^	0.00% (0/16)	0.00% (0/16)

*pWSSV1, standard WSSV plasmid.

**Figure 1:**
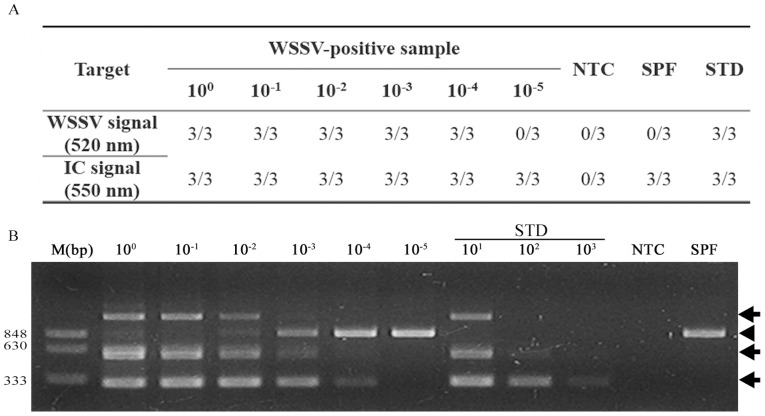
Sensitivity analysis of IQ Plus™ WSSV Kit with POCKIT System. WSSV-positive *L. vannamei* DNAs were diluted in 200 ng/ml genomic DNA prepared from SPF *L. vannamei* and subjected to analysis with IQ Plus™ WSSV Kit with POCKIT System (A) and WSSV IQ2000 PDS (B). The signals from WSSV and IC amplification were collected by the 520-nm and 550-nm channels, respectively. (B) Positive standard DNA provided by the kit was serially diluted (10^3^, 10^2^ and 10^1^ copies) as suggested by the manufacturer. Nested PCR Amplicons generated by WSSV IQ2000 PDS were analyzed by agarose gel electrophoresis and ethidium bromide staining. The kit was designed to generate three amplicons (arrows) from WSSV genome and one from host shrimp DNA (arrow head). Number of target amplicon bands correlates positively with the initial concentrations of target DNA template. NTC, negative control (ddH2O); SPF, DNA of SPF *L. vannamei*; STD, positive control plasmid; M, DNA weight molecular markers.

Furthermore, to compare the sensitivity of IQ Plus™ WSSV Kit with POCKIT system with that of IQ2000™ WSSV Detection and Prevention System (DPS), DNA extract prepared from a WSSV-positive *L. vannamei* was serially diluted using 10-fold increments in 20 ng/µl genomic DNA of SPF *L. vannamei* and subjected to analysis by both assays simultaneously. In iiPCR POCKIT assay, detection of the IC signals implied that both nucleic acid extraction and iiPCR steps were successful. Figure 1A shows that 550-nm IC signals were detected in all reactions containing shrimp DNA (Figure 1A, WSSV-positive sample and SPF). Among these, target WSSV signals (520 nm) were detected from 10^0^ to 10^4^ fold-diluted WSSV-positive samples. Detection limits of WSSV IQ2000 DPS were also reached at 10^−4^ dilution (Figure 1B), indicating that both assays had comparable detection limits to detect WSSV DNA in shrimp samples.

### IQ Plus™ WSSV Kit with POCKIT System does not Cross-react with Important Shrimp DNA Viruses

**Figure 2:**
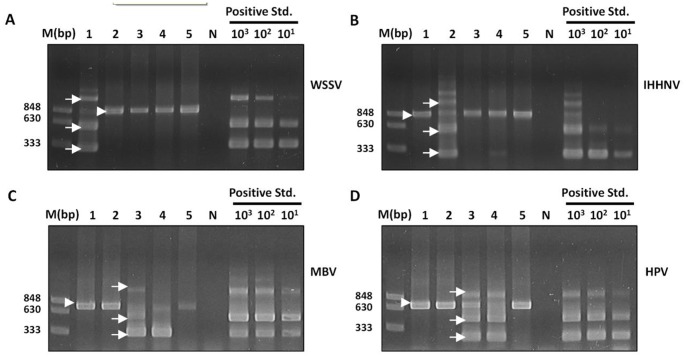
Specificity analysis of IQ Plus™ WSSV Kit with POCKIT System using WSSV, IHHNV, MBV, and HPV-positive samples. Genomic DNA extracts of diseased and healthy shrimp samples were subjected to analysis by IQ2000™ WSSV (A), IHHNV (B), MBV (C), and HPV (D) DPS assays, as well said Plus™ WSSV Kit with POCKIT system. Positive standard DNA provided by the kit was serially diluted (10^3^, 10^2^ and 10^1^ copies) as suggested by the manufacturer. Nested PCR amplicons were analyzed by agarose gel electrophoresis and ethidium bromide staining. The kits were designed to generate three amplicons (arrows) from target viral genome. The number of the amplicon bands positively correlates with the concentrations of the starting DNA template. The same set of samples was analyzed by IQ Plus™ WSSV Kit with POCKIT System analysis (E). M, DNA weight molecular markers (bp); N, negative control (ddH_2_O); Std, standard plasmid.

In order to assess the specificity of the IQ Plus™ WSSV Kit with POCKIT system assay, *L. vannamei* infected strongly with important shrimp DNA viruses, including WSSV (sample No.1), IHHNV (infectious hypodermal and haematopoietic necrosis virus, sample No. 2), MBV (*monodon* baculovirus, sample No. 3), and HPV (hepatopancreatic parvovirus, sample No. 4), were selected by nested PCR-based IQ2000 assays (Figure 2A–2D). A healthy shrimp was also included (sample No. 5). The same set of samples was tested using IQ Plus™ WSSV Kit with POCKIT system (Figure 2E). IC signals, but no WSSV signals, were detected from the healthy shrimp. WSSV signals were detected only from the WSSV-positive but not from IHHNV-, MBV- or HPV-positive samples, which were also all tested IC positive (550 nm), suggesting that IQ Plus™ WSSV Kit with POCKIT system did not cross-react with these shrimp DNA viruses nor with host genome.

### Precision Test

To evaluate intra-assay and inter-assay repeatability of IQ Plus™ WSSV Kit with POCKIT system, DNA extracted from one WSSV-negative and two WSSV-positive *L. vannamei* samples were analyzed in four replicates by two operators on two days. Table 2 shows that 100% agreement was found within and between tests of both WSSV (520 nm) and IC (550 nm) for each sample, demonstrating high precision of IQ Plus™ WSSV Kit with POCKIT system for detection of WSSV DNA in shrimp samples.

**Table 2:** Intra- and inter-assay repeatability of IQ Plus™ WSSV Kit with POCKIT system.

		Operator no. 1			Operator no. 2		
Day	Lot no.	Sample no.			Sample no.		
		1	2	3	1	2	3
1	1	0/4	4/4	4/4	0/4	4/4	4/4
1	2	0/4	4/4	4/4	0/4	4/4	4/4
1	3	0/4	4/4	4/4	0/4	4/4	4/4
2	1	0/4	4/4	4/4	0/4	4/4	4/4
2	2	0/4	4/4	4/4	0/4	4/4	4/4
2	3	0/4	4/4	4/4	0/4	4/4	4/4
3	1	0/4	4/4	4/4	0/4	4/4	4/4
3	2	0/4	4/4	4/4	0/4	4/4	4/4
3	3	0/4	4/4	4/4	0/4	4/4	4/4

### Accuracy Test

IQ Plus™ WSSV Kit with POCKIT system was compared with IQ2000™ WSSV DPS for its accuracy in detecting WSSV DNA in shrimp samples. Sensitivity and specificity were determined according to the agreement between the two assays using positive and negative samples, respectively. First, 400 positive (including at least 100 lightly infected specimens) and 300 negative reference animals identified previously by IQ2000™ WSSV DPS (data not shown) were tested in parallel by both IQ Plus™ WSSV Kit with POCKIT system and IQ2000™ WSSV DPS. Results are summarized in Table 3. Kappa statistics performed to generate 95% confidence intervals indicates sensitivity: 93.5% [95% confidence interval (CI): 90.61–95.56%] and specificity: 97.0% (95% CI: 94.31–98.50%).

**Table 3:** Accuracy test of IQ Plus™ WSSV Kit with POCKIT system using defined WSSV-positive and WSSV-negative *L. vannamei* samples.

		IQ2000™ WSSV DPS		
		Positive	Negative	Total
**IQ Plus™**	Positive	374	9	383
**WSSV Kit with**	Negative	26	291	317
**POCKIT system**	Total	400	300	700

Furthermore, 100 un-defined shrimps obtained randomly from a local farm were tested. Table 4 shows that 100% agreement was observed between IQ2000™ WSSV DPS and IQ Plus™ WSSV Kit with POCKIT system for both sensitivity and specificity, with 19% of the samples tested positive by both assays.

**Table 4:** Accuracy test of IQ Plus™ WSSV Kit with POCKIT system using undefined *L. vannamei* samples.

		IQ2000™ WSSV DPS		
		Positive	Negative	Total
**IQ Plus™**	Positive	19	0	19
**WSSV Kit with**	Negative	0	81	81
**POCKIT system**	Total	19	81	100

### Proficiency Test

Proficiency tests that involved three laboratories were performed to assess inter-lot, inter-laboratory and inter-operator reproducibility. Aliquots of 64 shrimp samples and three different batches of IQ Plus™ WSSV Kit with POCKIT system were shipped to the laboratories and repeated independently by two operators at each site. Six test results were obtained for each sample and analyzed by Chi-square test and Fisher’s exact test for homogeneity evaluation of the kit. A total of 384 tests were done in each laboratory. Little heterogeneity was found among these three laboratories (χ^2^ = 0.54 and P = 0.76, Chi-square test; P = 0.81, Fisher’s exact test). In addition, no significant difference was found among lots (Table 5) nor operators (Table 6), indicating the established kit performed with great reproducibility.

**Table 5:** Analysis of inter-lot reproducibility of IQ Plus™ WSSV Kit with POCKIT system.

Labs	Lots	DF	Total number	χ^2^	P-value (χ^2^)	P-value*
A	3	2	384	1.70	0.43	0.53
B	3	2	384	0.42	0.81	0.95
C	3	2	384	0.11	0.95	1

DF, degree of freedom.

*Fisher’s exact test.

**Table 6:** Analysis of inter-operator reproducibility of IQ Plus™ WSSV Kit with POCKIT system in three laboratories.

Labs	Operators	DF	Total tests	χ^2^	p-value (χ^2^)	p-value*
A	1,2	1	384	0	1	1
B	1,2	1	384	0.62	0.43	0.60
C	1,2	1	384	0.06	0.81	1

DF, degree of freedom.

*Fisher’s exact test.

## Discussion

In this study, extensive evaluation and validation studies were performed to demonstrate the sensitivity, specificity, precision and accuracy of IQ Plus™ WSSV Kit with POCKIT system. Test results show that this system performed with great analytical sensitivity and specificity. The assay could detect as low as 17 copies of WSSV genomic DNA per reaction (95% LOD) and reach detection limits similar to those of the nested PCR-based IQ2000™ WSSV DPS, an OIE-registered WSSV detection assay. Furthermore, IQ Plus™ WSSV Kit with POCKIT system did not cross-react with other important shrimp DNA pathogens, including IHHNV, MBV, and HPV.

The POCKIT™ device is developed to detect and measure fluorescent signals generated during PCR amplification to generate signal/noise (S/N) ratios. The S/N ratios are grouped into positive or negative readouts according to the built-in thresholds. Compared to OIE-registered IQ2000™ WSSV DPS, IQ Plus™ WSSV Kit with POCKIT system showed similar diagnostic accuracy on the basis of 700 prescreened *L. vannamei* with known WSSV infection status, *i.e.* sensitivity: 93.5% [95% CI: 90.61–95.56%] and specificity: 97.0% [95% CI: 94.31–98.50%]) in this study. In addition, analysis of 100 randomly selected samples shows the established assay had 100% sensitivity and specificity. Therefore, positive and negative readouts derived from the built-in S/N threshold correlated well with the results from IQ2000™ WSSV DPS, supporting that the default S/N thresholds in POCKIT™ for fluorescent probe-based iiPCR have been set properly.

This is the first report with rigorous validation studies to demonstrate that an assay based on the newly developed iiPCR POCKIT system could detect WSSV in shrimp sample with accuracy similar to conventional nested PCR. Compared to IQ2000™ WSSV DPS, different degrees of agreement were found in tests using 700 previously defined and 100 random samples for IQ Plus™ WSSV Kit with POCKIT System (Tables 3 and 4). Sensitivity of 93.5% and specificity of 97.0% were found from the analysis of 700 previously defined samples (Table 3), whereas100% agreement for both sensitivity and specificity was observed with 100 randomshrimp samples (Table 4). The minor discrepancy found between the results of these two tests could be attributed to different sampling sizes and possible variations within sampling population, such as levels of infection. According to test results of IQ2000™ WSSV DPS, the previously defined and random populations were composed of different percentage of WSSV-negative samples (42.9% and 81%, respectively). Although validation tests were performed only with *L. vannamei* samples in this study, positive IC signals were also obtained from *P. monodon* (data not shown). Even though the 18S rRNA gene is conserved among crustaceans, whether the IC reaction in IQ Plus™ WSSV Kit with POCKIT system would work with a particular host should be evaluated before the assay is to be used for WSSV screening.

The IQ Plus™ WSSV Kit with POCKIT system requires simple steps and could be completed in one hour in a portable POCKIT™ device without any post-amplification sample processing. Assembly of the reaction involves 3 steps, namely rehydration of the lyophilized reagents, addition of sample nucleic acids, and transfer of reaction mixture into reaction vessels. Automatic readouts generated with the default algorithm of POCKIT™ are shown on the screen immediately after reaction completes, eliminating any manual data processing step to allow the assay to be performed by anyone with basic protocol training. In combination with a quick nucleic acid extraction kit, it takes less than 90 min from sample to result. However, the IQ Plus™ WSSV Kit with POCKIT system does require specialized reagents and equipment, including a mini-centrifuge and the POCKIT™ device, to perform.

A variety of PCR-based assays have been developed for WSSV detection [7], [14], [15], [16], [17], [18], [19], [20], [21], [22]. The requirements of either post-amplification process (conventional PCR) or sophisticated and expensive machine (real-time PCR) have limited their application to only laboratories with adequate equipment and trained technicians. In order to meet the requirements of a point-of-need WSSV detection assay, an iiPCR assay based on fluorescent probe hydrolysis was demonstrated to detect WSSV sensitively and specifically [10]. However, it was a singplex reaction performed in a prototype iiPCR device, which could process signals through only one optical channel (520 nm) [10]. The recently launched POCKIT™ included an optical system to detect 520-nm and 550-nmsignals separately to allow multiplex iiPCR, based on the prototype iiPCR device. The multiplex WSSV iiPCR described herein is the first assay to take full advantage of the POCKIT™ device.

Suboptimal nucleic acid extraction and iiPCR conditions could lead to false-negative results. The IC reaction included in IQ Plus™ WSSV Kit with POCKIT system targets the shrimp’s nuclear18S rRNA gene, enabling users to monitor performance of the nucleic extraction and iiPCR reaction steps. Accordingly, the presence of IC signal and the absence of target signal indicate that WSSV is not detectable in the samples. The absence of IC signal alerts users that problems have occurred at DNA extraction and/or iiPCR stages, mitigating risks of obtaining false negative results.

Recently, various loop-mediated isothermal amplification (LAMP) assays have been developed for WSSV detection [23], [24], [25], [26], [27]. Requiring a simple incubator, LAMP also has the potential to be applied to point-of-need detection of aquaculture pathogens [28], [29]. WSSV LAMP assays generally complete within one hour at around 63°C and have sensitivity limit of approximately 10 to 100 copies of template DNA. However, it is in general more challenging to optimize and multiplex LAMP assays because they require four to six primers to hybridize with six to eight areas in one target area. Detection of target amplicon-specific signals would reduce greatly the risks of false positive results derived from non-specific LAMP amplicons [23], [26], [30], [31]. However, to our best knowledge, portable devices for target amplicon-specific detection are not available currently for field application of LAMP assays.

WSSV-like sequences, occupying around 20% of the *P. monodon* genome, has been found to be present throughout the shrimp genome in a genome sequencing study [32]. This is a cause for concern that the probability of false-positive diagnosis of white spot syndrome disease is likely to rise from cross reactivity of PCR primer and probe with target homologs within shrimp genome. Rigorous sequence BLAST analysis of the WSSV target sequence has been regularly carried out against sequences available in GenBank database, including all crustaceans. Furthermore, sequences of significant similarity with the target sequence have not been found in the *P. monodon* genomic sequences reported by Huang et al., 2011. Possible false positive detection of WSSV due to such sporadic DNA recombination events will be monitored continuously in the field, to alert us to implement proper adjustment of the assay in time.

High through-put real-time PCR assay or gel-based conventional nested PCR assays are recommended for laboratories staffed with skilled technicians and required to process large numbers of samples for disease diagnosis and surveillance purposes. These methods could be afforded mostly by large-scale shrimp farms, and government-supported or commercial diagnostic laboratories. Shipping samples to be tested at centralized laboratories usually results in delayed responses and extra costs, especially for facilities at remote areas. The IQ Plus™ WSSV Kit with POCKIT system, a diagnosis assay allowing pond-side detection of WSSV, would help shrimp farmers and local offices to respond to disease outbreaks in an efficient and timely manner. For field users, equipment and accessories required to run the assay (POCKIT™, a mini-centrifuge, pipettes, and pipette tips)are combined into a mobile package (POCKIT™ Xpress) to allow great mobility of the system. Compared to shipping samples to centralized laboratories for WSSV diagnosis, the POCKIT™ assay could significantly lower the costs and shorten the sampling-to-result turn-around time from days to a few hours. Testing by IQ Plus™ WSSV Kit with POCKIT system costs around US$10 per sample, which is relatively inexpensive in comparison to the costs of sending samples to be diagnosed by standard and/or real-time PCR assays at a laboratory, which could cost more than US$50 for each target pathogen plus fees for handling and shipping.

Allowing eight reactions per run, the field-deployable POCKIT™ system is designed to fulfill the need of small-scale or branch facilities where, with a good sampling plan, high sample throughputs are not required. Furthermore, sensitivity of the POCKIT™ system makes it possible to detect WSSV at early stages, enabling users to take appropriate bio-security measures in a timely manner. In addition, the POCKIT™ system would facilitate on-site investigation of WSSV outbreaks, where relatively low numbers of known diseased animals are required to be tested.

Great reproducibility in WSSV detection by the IQ Plus™ WSSV Kit with POCKIT system was observed among the results generated by six operators at three laboratories, indicating excellent repeatability of the assay. This could be attributed partly to that the kit is available in a lyophilized format, which ensures stability of the reagents during the shipping and storage stages. Therefore, IQ Plus™ WSSV Kit assay has the potential to serve as a rapid, specific and sensitive tool for routine point-of-need detection of WSSV for shrimp farming industry.
